# Cost-effectiveness of facility-based, stand-alone and mobile-based voluntary counseling and testing for HIV in Addis Ababa, Ethiopia

**DOI:** 10.1186/s12962-020-00231-x

**Published:** 2020-09-11

**Authors:** Amanuel Yigezu, Senait Alemayehu, Shallo Daba Hamusse, Getachew Teshome Ergeta, Damen Hailemariam, Alemayehu Hailu

**Affiliations:** 1 School of Public Health, Addis Ababa University, Addis Ababa, Ethiopia; 2 Bergen Center for Ethics and Priority Setting, Department of Global Public Health and Primary Care, University of Bergen, Bergen, Norway; 3 Ethiopian Public Health Institute, Addis Ababa, Ethiopia; 4 Federal Ministry of Health, Grand Challenge Ethiopia, Addis Ababa, Ethiopia

**Keywords:** Ingredients costing, Decision tree modeling, Reciprocal cost allocation, Cost-effectiveness analysis, VCT models

## Abstract

**Background:**

Globally, there is a consensus to end the HIV/AIDS epidemic by 2030, and one of the strategies to achieve this target is that 90% of people living with HIV should know their HIV status. Even if there is strong evidence of clients’ preference for testing in the community, HIV voluntary counseling and testing (VCT) continue to be undertaken predominantly in health facilities. Hence, empirical cost-effectiveness evidence about different HIV counseling and testing models is essential to inform whether such community-based testing are justifiable compared with additional resources required. Therefore, the purpose of this study was to compare the cost-effectiveness of facility-based, stand-alone and mobile-based HIV voluntary counseling and testing methods in Addis Ababa, Ethiopia.

**Methods:**

Annual economic costs of counseling and testing methods were collected from the providers’ perspective from July 2016 to June 2017. Ingredients based bottom-up costing approach was applied. The effectiveness of the interventions was measured in terms of the number of HIV seropositive clients identified. Decision tree modeling was built using TreeAge Pro 2018 software, and one-way and probabilistic sensitivity analyses were conducted by varying HIV positivity rate, costs, and probabilities.

**Results:**

The cost of test per client for facility-based, stand-alone and mobile-based VCT was $5.06, $6.55 and $3.35, respectively. The unit costs of test per HIV seropositive client for the corresponding models were $158.82, $150.97 and $135.82, respectively. Of the three models, stand-alone-based VCT was extendedly dominated. Mobile-based VCT costs, an additional cost of USD 239 for every HIV positive client identified when compared to facility-based VCT.

**Conclusion:**

Using a mobile-based VCT approach costs less than both the facility-based and stand-alone approaches, in terms of both unit cost per tested individual and unit cost per HIV seropositive cases identified. The stand-alone VCT approach was not cost-effective compared to facility-based and mobile-based VCT. The incremental cost-effectiveness ratio for mobile-based VCT compared with facility-based VCT was USD 239 per HIV positive case.

## Background

HIV is one of the most devastating global epidemics in human history. Since the beginning of the epidemic, about 78 million people had been infected, and more than 35 million people have died. Globally, in 2016, about 42 million people are living with HIV/AIDS [1], out of which, 19.4 million are living in Eastern and Southern African regions. The number of new HIV infections in 2016 was 1.8 million worldwide, and sub-Saharan African accounts for three-quarters of the new infections and deaths [2]. In Ethiopia, about 27,200 new HIV infections happened in 2016, and the incidence of infection in the urban areas (6.8%) is many times higher than in rural areas (0.7%) [3].

UNAIDS’s 90-90-90 goal sets new targets. This aim of this goal is to detect 90% of people living with HIV by 2020, to treat and retain 90% of those who are identified as HIV positive on antiretroviral therapy (ART), to reduce the viral load to an undetectable level for 90% of those on ART [4]. Evidence shows that if the 90-90-90 goal is achieved by 2020, it will help end the epidemic by 2030, which will have profound economic and health benefits [5]. To ensure timely access to effective HIV treatment and reinforce the prevention of new infections, the creation of awareness of HIV status through HIV voluntary counseling and testing (VCT) is a crucial activity [6].

Among women and adults between the ages of 15–49, the proportion of people who have been tested for HIV is about 70% globally and 50% in Africa [7]. Similarly, more than half of the Ethiopian population, in general, and 27% of people living in the capital city, Addis Ababa, had never been tested, and only 67% of HIV seropositive individuals know their HIV status [8]. However, the target set by UNAIDS is to detect 90% of HIV positive individuals by the end of 2020. To reach this ambitious goal, a cost-effective VCT model that identifies more HIV positive individuals is required.

In Ethiopia, the current HIV testing and counseling (HTC) service delivery models are classified into four: integrated facility-based HTC services (Facility-based VCT and provider-initiated testing and counseling), Standalone VCT, Outreach, and mobile VCT, and Workplace HTC service. The services are generally established on community-based and facility-based HIV testing and counseling approaches [9]. Facility-based VCT is HIV testing and counseling conducted in health facilities (hospitals, health centers, and private clinics) initiated by the clients. Stand-alone-based VCT is a type of HIV counseling and testing service delivery model conducted outside hospitals and health centers as a fixed VCT operating on its own. Mobile-based VCT is a type of community-based HCT conducted by setting up a mobile van or container to provide HIV counseling and testing services in a community’s central area. The clients initiate all three testing strategies. Their difference is in the place of service delivery. Facility and stand-alone-based VCT are services provided at specific places, whereas mobile-based VCT provides services in the community’s central area. The facility, stand-alone, and mobile-based VCT models followed standardized procedures for service delivery. VCT is free, voluntary, and confidential and is delivered by trained counselors after a serial algorithm of rapid HIV antibody tests with a finger-prick blood sample collection [10].

The unit cost per client tested for HIV at the facility-based VCT is USD 5 in Malawi, and USD 4 in Zambia, USD 9 in Zimbabwe [11], and ranges from USD 5.05 to 16 in Kenya [12]. The cost per client tested for HIV for the stand-alone-based VCT is USD 20 in a systematic review of low and middle-income countries [13], USD 51 in South Africa [14], USD 19 in Uganda [15], USD 58 in Vietnam [16], and USD 60 in Namibia [17]. The cost per client tested for HIV in a mobile-based VCT is USD 25 South Africa, USD 15 in Kenya and USD 60 in Namibia [17–19]. A systematic review conducted also estimated the cost per client test through mobile-based VCT to be USD 60 [20].

Some studies show that, although the costs tend to be higher than facility-based HTC services, mobile-based HCT is better to reach HIV infected individuals earlier in the disease progression [21–23]. Mobile-based HCT detects more first-time testers and HIV positive individuals with high CD4 cell counts. Community-based HTC with mobilization and enhanced linkage to care can overcome barriers to HIV testing and linkage to care, achieving widespread coverage of testing and antiretroviral therapy [24, 25]. Community-based HTC also relies less heavily on existing infrastructure, allowing easier scale-up [20]. Currently, the VCT service continues to be undertaken predominantly in health facilities [26]. However, evidence shows that less time and costs are spent by the people who use mobile-based VCT than the facility-based VCT [27, 28]. Besides, clients prefer more to be tested in the community than in health facilities [29, 30].

The choice of different HIV testing and counseling models should be based on evidence about the total costs of the services and the total health benefit accrued from each of the strategies. The costs and the health benefit are affected by different factors, such as physical and financial accessibility of the services, HIV prevalence, the country’s economic status, the skill of counselors, the degree of emphasis placed on careful and intensive counseling, the number attending relative to the capacity of the service, the type of test and number of tests [13, 31, 32]. Country-level cost-effectiveness evidence about different HIV counseling and testing models are important to inform priority-making decisions. However, none of the studies conducted in Ethiopia tried to investigate this. Therefore, this study aimed to compare the cost and cost-effectiveness of facility-based, stand-alone and mobile-based HIV voluntary counseling and testing in Addis Ababa, Ethiopia.

## Methods

### Study setting

This study was conducted in Addis Ababa, Ethiopia’s capital city, with a total population of about 3.4 million. There are 11 hospitals, 97 health centers, one stand-alone VCT clinics, and 15 mobile VCT service sites in the city. The city has the highest concentrations of HIV/AIDS cases in the country, with an estimated prevalence of 4.9% and contributes 16% of the country’s new infection in 2016 [3].

### Sampling and data collection

Primary cost data were collected from July 2016 to June 2017 from selected facilities in Addis Ababa city. For facility-based VCT costing, from 97 health centers providing VCT service in the city, only ten health centers were included due to budget constraints. One health center was randomly included from each sub-city, and only facility-based VCT is considered and not the provider-initiated HTC. For stand-alone and mobile-based testing, a center-specific costing approach was applied. In Addis Ababa, there is only one stand-alone VCT testing facility, and we include that facility in this study. There are 15 mobile-based VCT centers in the city, and all of them were included in the costing exercise. Data for the stand-alone and mobile testing sites were collected from the AHF.

### Costing approach

This study uses an ingredient costing approach to determine costs whereby actions to be taken under intervention are listed, specific resources needed to implement the intervention are described, and costs are assigned to all the resources based on opportunity costs used for the intervention [33, 34]. It allows analysts from one country to assess if costs collected in another country can be used or modified to their settings [35]. However, the use of the ingredients costing approach makes it necessary to determine overhead costs. The complementary cost allocation approach was used to estimate the overhead costs. It fully recognizes complementary services provided among all support service centers and allocate costs between the support service centers [36]. All the costs were adjusted for inflation using a consumer price index of the year 2019 as a base year cost. All costs were expressed in 2019 US Dollars.

Capital costs include buildings, equipment, and vehicles, which were annualized using a discount rate of 5% with an assumed lifespan of 30 years for vehicles and 5 years for equipment [32, 37]. Because some buildings are older than their expected year of service, others are rented and recently built; the rental cost of a building was considered an economic cost to have the same cost comparison across the interventions. Recurrent costs include personnel, gloves and test kits, other supplies, vehicle operation and maintenance, and building operation and maintenance. About 10% of the annual rent cost was used as the annual cost of building operation and maintenance [32] (Table 1).

**Table 1:** Recurrent and capital cost components of the VCT service costing

Recurrent cost	Allocation base	Cost data	Method of data collection and data source
Personnel	Time worked	Total payment for full-time staff, part-time staff, and volunteers	Review of annual payroll, and reports and interview
Supplies	Weight/volume	Replacement value	Review of activity, reports, and interview
Operation and maintenance of a vehicle	Time used and volume	Replacement value	Review of reports and interview
Capital cost		Working life year (source)	
Buildings	Space used	Rental value	Interview
Equipment	Time used	Replacement value and annualization [32, 37]	Review of fixed assets list and interview
Vehicles	Time used	Replacement value and annualization [32, 37]	Gov’t contracts, supply record from NGO, local dealers
Others		Actual value	

### Cost allocation method

The service sites were health centers (facility-based VCT), stand-alone VCT clinics, and mobile VCT sites. The service sites were divided into the care service center (VCT center) and support service center. Support service centers were divided into ancillary and site management. The ancillary support service centers include the cleaning and transportation sections. The site management includes site administration, security, finance, human resources, disease prevention, medical director, counselor coordinator, country program director, accountant, and data clerk.

All the capital and recurrent inputs of the support service centers were identified, measured, and valued. The total cost for each support service center was then calculated. Costs were allocated among the support service centers using the reciprocal cost allocation method. We took into account the number of staff per service output of the support service centers as the allocation base. After allocating the costs within the support service centers, allocating the costs from support service centers to the care service centers (VCT) was made.

The cost allocated from the support service center is added to the VCT center. Since the resource use (e.g., counselor’s time and test kits) to identify HIV-positive and HIV-negative client is different, we used the time it takes for testing and the number of tested and positive individuals as the allocation base to estimate the cost for tested and HIV positive clients. The total amount of time per client was estimated by interviewing counselors on the time they spent to identify HIV positive and HIV negative test result, pre-test counseling time, testing time, and post-test counseling time with the patients. The number of tested clients and HIV positive clients were collected from the facility record. The unit costs and the total costs of the VCT service site was then calculated [38, 39].

Overall, the cost analysis included in this study were unit cost per positive-case, cost per test, the total capital cost per tested-case, recurrent cost per tested-case. Furthermore, descriptive analysis was performed by calculating the number and percentage of clients tested for HIV and HIV positive clients by age and sex for the three HIV testing approaches.

### Cost-effectiveness model

**Fig. 1:**
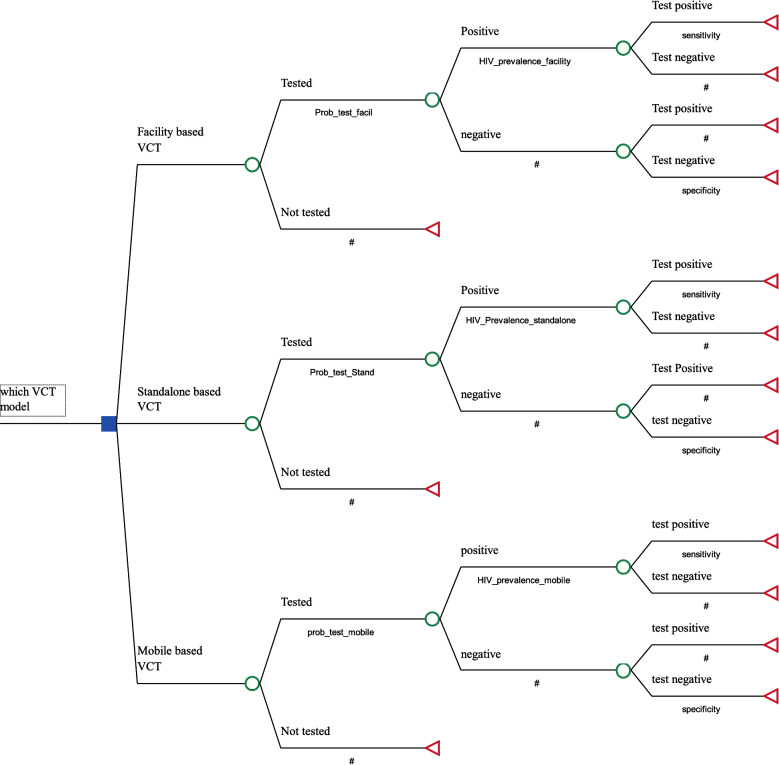
Model structure of the cost-effectiveness study

A decision tree model was built using TreeAge Pro 2018 software [40]. The decision tree model is the model’s choice when an event happens in a short period and does not happen repeatedly. The model follows a series of steps to construct a tree structure under uncertainty for alternative options and select the least expected cost per effect as the best alternative. A cost-effectiveness ratio was calculated for each of the VCT approaches using cost per HIV seropositive client tested as payoffs. The structure of the model of the cost-effectiveness study is presented in Fig. 1.

The decision to be addressed is “which VCT model is cost-effective in diagnosing HIV positive client among facility-based VCT, Stand-alone VCT and Mobile-based VCT,” expressed on the decision node at the start of the tree. The range of possible pathways that characterize the three testing strategies is explained on the chance nodes following the decision pathways. The pathways are built up through a series of branches representing the natural process of the testing event. Here the events are HIV testing, positivity rate, and test positivity. Branch probabilities are the likelihood of events issuing from a chance node and representing the possible events happening in the testing process at that point in the tree. Here the probabilities are the probability of being tested, the probability of being HIV positive, and test sensitivity. The combination of the different branches in the tree determines a series of pathways along which the HIV testing and counseling can pass in the tree. These pathways are mutually exclusive and exhaustive. So, probabilities at a pathway should sum to 100%. The analysis was performed through chance nodes showing uncertain previous events moving from left to right using conditional probabilities. Expected values in cost and effectiveness are then calculated. The three testing strategies’ expected cost is calculated by weighing each pathway cost by its respective probability and then summing across all the pathways. This decision model uses the probability of identifying HIV positive clients as the relevant measure of effect in the CEA (i.e., the probability of diagnosing HIV). In terms of expected effectiveness, this is equivalent to giving HIV diagnosis though testing the value = 1 and all other pathways, the value = 0 (i.e., HIV diagnosis = 1 and No diagnosis = 0). Following this, the expected effect is calculated by weighing each pathway’s effectiveness by its respective probabilities and then summing across all the pathways.

The input parameters presented in Table 2 for the tree structure are taken from literature and primary data from the health facilities. For example, the probability of being tested at a VCT site is calculated by dividing the number of people tested at a site by the estimated population of age above fifteen to be served under that service site. The urban health center serves 40,000 people, and the population age group of above 15 is around 57% of the total population [41]. The number of people tested for HIV in 2016, using each of the testing models, was divided by mid-year total population above 15 years of age. HIV disease prevalence was used to calculate the probability of being positive. To estimate the HIV positivity rate at the three testing modalities, we divided the number of HIV positive identified in a year by the total number of people tested for HIV for each testing modalities. Therefore, HIV positivity rates are different for each modality as the number of HIV positive individuals identified, and numbers tested for HIV are different for the three modalities. Finally, test sensitivity and test specificity were used for the actual status of the client.

**Table 2:** Input parameters of the cost-effectiveness model

Input parameters	Base value	Low value	High value	SD	Distribution	Data sources
Cost of positive for facility-based VCT	158.82	127.06	190.59	10.00	Gama	Primary
Cost of negative for facility-based VCT	4.95	3.96	5.94	0.49	Gama	Primary
Cost of positive for stand-alone VCT	150.97	120.78	181.17	10.00	Gama	Primary
Cost of negative for stand-alone VCT	6.39	5.11	7.67	0.64	Gama	Primary
Cost of positive for mobile-based VCT	135.53	108.42	162.63	10.00	Gama	Primary
Cost of negative for mobile-based VCT	3.28	2.63	3.9	0.32	Gama	Primary
Probability of being tested at facility-based VCT	0.050	0.030	0.100	0.006	Beta	Primary
Probability of being tested at stand-alone VCT	0.120	0.080	0.160	0.020	Beta	Primary
Probability of being tested at mobile-based VCT	0.340	0.200	0.400	0.080	Beta	Primary
Test sensitivity	0.997	0.993	1.000	0.001	Beta	[42]
Test specificity	0.992	0.990	1.000	0.002	Beta	[42]
HIV positivity rate: facility VCT	0.032	0.022	0.042	0.001	Beta	Primary
HIV positivity rate: stand-alone VCT	0.043	0.033	0.053	0.001	Beta	Primary
HIV positivity rate: mobile-based VCT	0.025	0.015	0.035	0.001	Beta	Primary

### Model assumption

Since there was no information on the number of people expected to be served under a single stand-alone and mobile VCT site, a common denominator of the catchment population based on the Ethiopian health care delivery system of the facility-based VCT (health center) for stand-alone and mobile-based VCT was used.

### Sensitivity analysis

Two types of sensitivity analyses were performed to deal with uncertainties in this study. First, a one-way sensitivity analysis was done using a tornado diagram for the low and high values of costs, probabilities, test-sensitivity, test-specificity and prevalence from the base case [43]. One-way sensitivity analysis was also conducted on HIV positivity rate, considering that HIV positivity rate is likely to change over time. The lower and the higher value choice were made considering the clinical and economic feasibility of the range concerning the setting. Second, probabilistic sensitivity analysis (PSA) was conducted to distribute the parameters used for a one-way sensitivity analysis. PSA uses a distribution rather than a predetermined value for each parameter. Gamma and beta distributions were used for cost and probabilities, respectively. The distribution for cost was varied by reviewing the cost of supply agencies from whom government health centers are expected to buy and allow variation of salary by looking at the least and highest salaries of the health facilities. Therefore, a 20% variation from the mean cost was allowed for the three models’ costs.

## Results

### Demographic characteristics of clients

The number of clients tested for HIV from July 2016 to June 2017 in the ten health centers, one stand-alone and 15 mobile sites was 12,913, 3155 and 128,199, respectively. The proportion of women tested for HIV was higher in the health centers (59%) and less in the stand-alone clinic (47%) and mobile sites (34%) than men. Of the clients tested, a vast majority were in the age group of 25–49 (Table 3).

**Table 3:** Clients who received VCT service from July 2016 to June 2017

Characteristics	Facility-based	Stand-alone	Mobile-based
Sex			
Male (positive)	5347 (134)	1685 (54)	85,668 (1389)
Female (positive)	7566 (278)	1470 (83)	42,214 (1780)
Age			
15–19	1897	430	10,267
20–24	3734	655	40,398
25–49	6468	1921	72,524
50+	814	149	4693
Total (positive)	12,913 (412)	3155 (137)	128,199 (3169)
Positivity rate	3.1%	4.3%	2.4%

Although a smaller number of women were tested in both stand-alone and mobile VCT sites than men, the proportion of positive cases was higher among females than in males. For example, from the total people tested and HIV positive cases, the proportion of females testing positive was 68%, 61%, and 58% in facility-based, stand-alone and mobile-based sites, respectively (Table 4). The proportion of females tested for HIV is higher in health centers than stand-alone and mobile-based testing for the age group 25-49. Female aged between 25 and 49 has a higher HIV positivity rate than male in the three testing approaches.

**Table 4:** Percent of tested and HIV positive clients by age from July 2016 to June 2017

Characteristics	Facility-based		Stand-alone		Mobile-based	
	Male	Female	Male	Female	Male	Female
Tested clients (%) by age group						
15–19	2.60	7.50	3.60	10.00	3.90	4.10
20–24	14.40	17.50	9.30	11.50	20.20	11.30
25–49	24.40	27.60	36.80	24.10	39.70	16.90
50+	2.70	3.20	3.80	1.00	3.00	0.70
Positive clients (%) by age group						
15–19	1.00	3.20	2.00	7.00	0.60	1.30
20–24	1.60	8.60	7.00	7.00	3.00	7.00
25–49	24.60	53.90	41.00	64.00	33.20	43.70
50+	4.40	2.80	4.00	5.00	7.10	4.10

### Costs per client tested

The cost per client test for facility-based VCT was USD 5.06 (SD 4.92, 5.21). Out of this, the cost of personnel account for 49% and the glove and test kit contribute to 30% of the cost. The cost of building, other supplies and equipment contributed to the rest of the cost. The cost per client tested for stand-alone VCT was USD 6.55. The cost of personnel and glove and test kit and building contribute to 54.4%, 22.4%, and 17.6% of the cost. Other supplies and equipment contribute to the remaining cost. The cost per client tested for mobile-based VCT was USD 3.35. Out of this, the cost of the glove and test kit, personnel, and vehicle rent contributes to 59%, 35.3%, and 9% of the cost, respectively. The building, other supplies, and equipment contribute to the remaining cost.

### Costs per HIV seropositive client

The costs per HIV seropositive for facility-based, stand-alone, and mobile-based VCT are presented in Table 5. The cost per test for HIV seropositive clients was USD 158.82 for facility-based VCT, USD 150.97 for stand-alone, and USD 135.52 for mobile-based VCT. Out of this, the cost of personnel and glove and test kit accounts for most of the costs. The contribution of cost from the support service center and cost from a direct service center to a single site for the three VCT models is presented in Table 5. The cost of the support service center was the highest for mobile-based VCT, followed by stand-alone and facility-based VCT.

**Table 5:** Total and unit cost of tested and test-positive by VCT types per HIV testing facility

Service site	Support center	VCT center	Total cost	Tested clients	Testing unit cost	Positive clients	Positive unit cost
Facility-based	1285.03	64,149.83	65,434.86	1291	5.06	41.20	158.82
Stand-alone	3633.09	17,050.24	20,683.34	3155	6.55	137.00	150.97
Mobile-based	3006.78	25,625.62	28,632.40	8546	3.35	211.26	135.52

### Cost-effectiveness ratio

The expected cost and effectiveness was calculated to determine the most cost-effective VCT model. Stand-alone VCT is extendedly dominated by mobile and facility-based VCT since the ICER of stand-alone-based VCT compared to facility-based VCT is higher than the next effective (mobile-based VCT) strategy. After excluding the extendedly dominated stand-alone-based VCT, the ICER of facility-based and mobile-based VCT was recalculated. The incremental cost-effectiveness ratio of the mobile-based VCT was USD 239 for the identification of additional HIV seropositive clients when compared to facility-based VCT (Table 6).

**Table 6:** Incremental cost-effectiveness ratios of facility-based, stand-alone, and mobile-based VCT

Strategy	Cost	Inc. cost	Effectiveness	Inc. effectiveness	ICER	ACER
Excluding dominated						
Facility-based VCT	0.53		0.002			269.87
Mobile-based VCT	2.72	2.19	0.011	0.009	239.01	244.51
All						
Facility-based VCT	0.53	0.00	0.002	0.000	0.00	269.87
Stand-alone-based VCT	1.67	1.14	0.006	0.004	279.34	276.24
Mobile-based VCT	2.72	1.05	0.011	0.005	206.50	244.51

*ACER* average cost-effectiveness ratio

### Sensitivity analysis

**Fig. 2:**
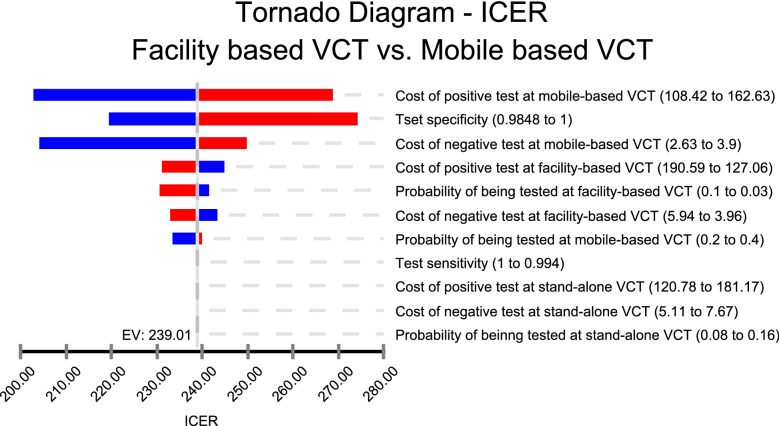
A tornado diagram

One-way sensitivity analysis using a tornado diagram with a least-likely and highly likely value of the selected variables is presented in Fig. 2. The tornado diagram indicates that the cost of a positive test at mobile-based VCT had the highest impact on the incremental cost-effectiveness ratio. Although the ICER is changing for lower and higher values of the cost of positive test at mobile-based VCT, the ICER showed around 17% change (from USD 158 to 225 per HIV seropositive client identified). The ICER was less sensitive to change in most of the other variables in general.

**Fig. 3:**
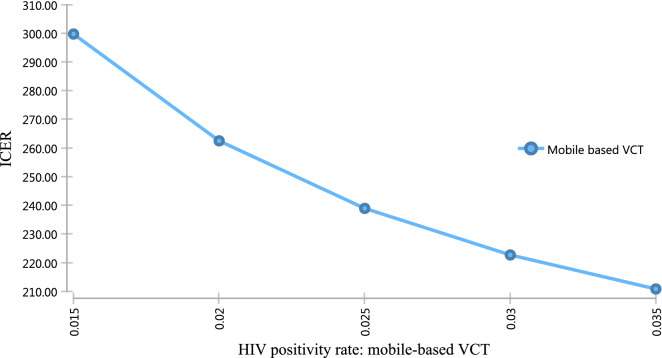
One-way sensitivity analysis of the HIV positivity rate at mobile-based VCT

As the cost of identifying HIV positive individuals increase at mobile-based VCT, the ICER also increases and vice versa. Therefore, mobile testing may be a more cost-effective approach in the high prevalence area (Fig. 3).

**Fig. 4:**
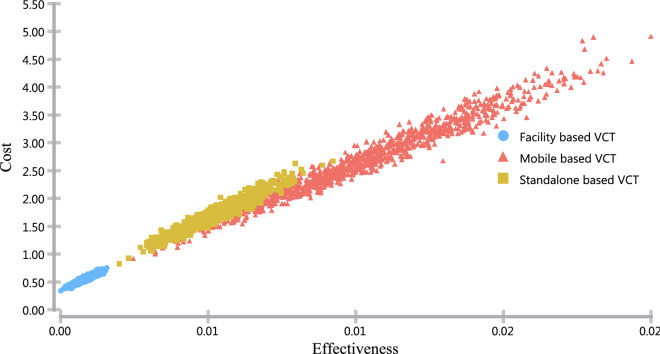
Cost-effectiveness scatter plot

**Fig. 5:**
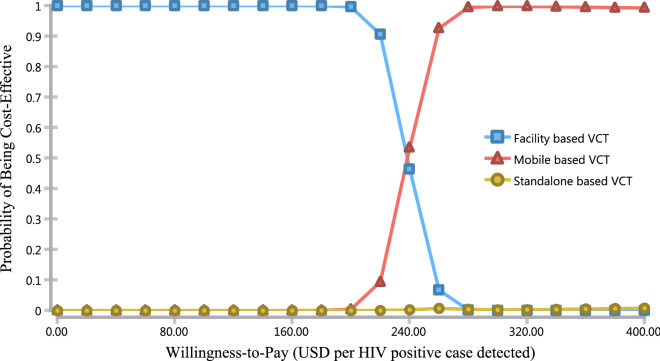
Cost-effectiveness acceptability curve

In Figs. 4 and 5, we present the probabilistic sensitivity analysis using the cost-effectiveness scatter plot and acceptability curve. The cost-effectiveness scatters plot indicates that there is less variability across both cost and effectiveness of facility-based VCT, while the variability in the other two options was vast in both cost and effectiveness dimension (Fig. 4).

The cost-effectiveness acceptability curves (CEAC) indicates the probability of being cost-effective at different levels of willingness to pay per HIV case detected. For example, the probability of being a cost-effective option for mobile-based VCT was about 10% at a willingness-to-pay threshold of USD 220, while at a willingness-to-pay threshold of USD 260, the probability of the mobile-based VCT being a cost-effective option was about 90% (Fig. 5).

## Discussion

This study is the first of its kind in Ethiopia to compare the cost-effectiveness of facility-based, stand-alone, and mobile-based voluntary counseling and testing.

### Cost of the VCT models

The unit cost per client tested for HIV at the facility-based VCT is USD 5.06. Our finding of cost per client tested using a facility-based VCT is very comparable estimated elsewhere. For example, a study in Malawi (USD 5) and Zambia (USD 4), but substantially lower than the finding Zimbabwe (USD 9) [11]. Similarly, studies in Kenya and Swaziland in 2012 also reported that the unit cost of facility-based VCT ranges from USD 5.05 to 16 and USD 8.6 to 19, respectively [12].

The cost per client tested for HIV for the stand-alone-based VCT is USD 6.55 (3155 clients tested). Although the number of clients tested is higher than the facility-based VCT, the unit cost is higher due to how the project cost is implemented. The cost per client test of stand-alone-based VCT in this study varies dramatically from other countries’ study reports. Studies of a systematic review of low and middle-income countries [13], South Africa [14], Uganda [15] Vietnam [16], and Namibia [17], showed the cost of stand-alone-based VCT estimated to be USD 20, 51, 19 and 58, and 60 per client tested respectively.

The cost per clients tested for HIV in a mobile-based VCT is USD 3.35 (8645 clients tested). The estimate of the cost per client through mobile-based VCT service under this study is lower than the cost estimate report in South Africa, Kenya, and Namibia with USD 25, 15, and 60 per client, respectively [17–19]. A systematic review conducted also estimated the cost per client test through mobile-based VCT to be USD 60 [20].

The cost per HIV seropositive client of facility-based, stand-alone and mobile-based VCT is USD 158.82, 150.97, and 135.52, respectively. The cost of HIV seropositive clients at a facility-based VCT is higher than in some study reports [11, 13, 15] and lower for study reports conducted in other countries [16, 17]. Some studies reports from other settings are comparable to our findings on the cost of HIV seropositive test for stand-alone [16, 19] and mobile-based VCT [13, 19], although it varies in another study [17]. The variation in the unit costs among various studies might be partly attributed to the costing method applied, and the resources accounted in each of the studies, the difference in HIV prevalence in the area [13], organizational structure, economy of the country, and the level of encouragement of the population to test for HIV which determines the level of economies of scale [31, 32].

This study is not without limitations. The measure of effectiveness is reported in intermediate outcomes with the number of clients tested and HIV seropositive identified. The use of intermediate outcome might pose difficulty compared with other interventions that are not reported with the same outcome measure. We did not use DALY measurement because of the lack of validated data on disability weights or one of the outcome measures. Since mobile-based VCT only performed in the year 2016, only a one-year data on clients were used for the three models. Some cost information was not available for the year the items were bought. To fill this gap, we estimated the costs by using the current market price of the items. A common denominator of the expected number of services provided at a health center is used for both stand-alone and mobile-based VCT to calculate the probability of being tested. A similar denominator is used because there is no standard set to determine the number of populations expected to be served under stand-alone and mobile-based VCT. Our data do not inform about repeated testers and new testers, which might influence the result if there is a repeated test for those HIV positive clients who knew their HIV status.

### Cost-effectiveness of the VCT models

Among the three models, stand-alone-based VCT was extendedly dominated by a linear combination of facility-based and mobile-based VCT. Therefore, it was excluded from the further calculation of the incremental cost-effectiveness ratios. We found that the incremental cost of mobile-based VCT to identify one more HIV seropositive client was USD 239 compared with facility-based VCT. Therefore, this study suggests that more HIV positive cases can be identified through a mobile-based VCT if some additional resources can be allocated to implement a mobile-based VCT service.

Currently, in Ethiopia, about 28% of the populations who are infected with the virus do not know their HIV status. The prevalence of HIV in an urban area is higher than the rural area, and it is known that the mobile-based VCT services are suited to highly populated areas [9, 20]. Providing VCT through mobile-based service can decrease the number of HIV positive individuals who do not know their HIV status, and therefore, can decrease the transmission of the virus. Besides, it will help to achieve the UNAIDS 90-90-90 target that infected individuals who do know their status are more likely to start ART early [4]. Other studies elsewhere, such as South Africa and Kenya, recommend that mobile-based VCT can be an essential and cost-effective approach in addition to the facility-based approach [18, 19].

The ICER of mobile-based VCT remains stable in all sensitivity analysis for the selected parameters. The ICER was similar to the base case result in lower and higher values and the changing distributions. The one-way sensitivity analysis indicated that as the prevalence of HIV increases, the mobile-based VCT would give more value for money. As the prevalence of HIV is 4.9% in Addis Ababa and other major cities, testing through mobile-based VCT can be a big opportunity [3]. Besides, the cities are relatively densely populated, and this makes them more suited for mobile-based testing [9, 20].

The probabilistic sensitivity analysis indicates that the incremental net monetary benefit of providing mobile-based VCT over the facility-based VCT increases linearly as the willingness to pay rises above USD 239. A recent report from UNAIDS indicates that about USD 6 billion is still needed to achieve the 90-90-90 goal. Supporting HIV prevention activities in developing countries is a continuous process, not a one-time action to eliminate it. In Ethiopia, the distribution of HIV is highly prevalent in urban areas. Interventions for a target population, such as female sex workers, are also made. Although this is a crucial step towards reducing transmission of the virus, HIV testing through the most efficient ways, such as the mobile-based VCT, is mandatory to reduce the overall prevalence in urban communities with a higher number of FSWs.

## Conclusion

Using a mobile-based VCT approach costs less than both the facility-based and stand-alone approaches in terms of unit cost per tested individual and unit cost per HIV seropositive cases. Stand-alone-based VCT was extendedly dominated by a combination of facility-based and mobile-based VCT approaches, and therefore, was not cost-effective. The incremental cost-effectiveness ratio for mobile-based VCT compared with facility-based VCT was USD 239 per HIV positive case identified.

## Data Availability

The datasets used and analyzed during the current study are available from the corresponding author on reasonable request.

## Abbreviations

AIDS: Acquired Immune Deficiency Syndrome
ART: Anti-retroviral therapy
CD4: Cluster of differentiation 4
CEA: Cost-effectiveness analysis
EFY: Ethiopian fiscal year
FSW: Female sex workers
HTC: HIV testing and counseling
HAPCO: HIV/AIDS Prevention and Control Office
ICER: Incremental cost-effectiveness ratio
INMB: Incremental net monetary benefit
OSSA: Organization for Social Service and Development
PICT: Provider Initiated Counseling and Testing
PSA: Probabilistic sensitivity analysis
SNNPR: Southern Nations, Nationalities, and Peoples’ Region
US: United States
UNAIDS: Joint United Nations Program on HIV/AIDS
USAID: United States Agency for International Development
VCT: Voluntary counseling and testing
WHO: World Health Organization
